# Association of Hormonal Contraceptives with Depression among Women in Reproductive Age Groups: A Cross-Sectional Analytic Study

**DOI:** 10.1155/2024/7309041

**Published:** 2024-09-20

**Authors:** Sadia Sultan, MD. Abu Bashar, Rahma M. Bazhair, Doaa O. Abdurahman, Renad A. Alrehaili, Meimouna E. Ennahoui, Yasmeen S. Alsulaiman, Seba D. Alamri, Elgawhara F. A. Mohamed

**Affiliations:** ^1^ Clinical Science Department-MBBS Program Fakeeh College for Medical Sciences, Jeddah, Saudi Arabia; ^2^ Department of Community and Family Medicine All India Institute of Medical Sciences, Gorakhpur, Uttar Pradesh, India

## Abstract

**Introduction:**

Hormonal contraceptives (HCs) are used for birth control, menstrual disturbances, and premenopausal syndrome. Most women stop using hormonal contraceptives due to changes in their mood. The evidence regarding the association of hormonal contraception with depression shows mixed results. Therefore, we aim to establish the association between the use of hormonal contraception and depressive symptoms.

**Methods:**

A cross-sectional study was conducted on 326 women of the reproductive age group (15–49 years) attending the family planning unit of the obstetrics and gynecology department of a medical college in Saudi Arabia. Their sociodemographic and medical details along with the current use of any contraceptives (hormonal, nonhormonal, or not using any) with duration were collected. Beck depression inventory-II (BDI-II) was applied to the women to assess for depression along with its severity, and a BDI score of >16 was taken to denote clinical depression. Women were stratified by type of contraceptive used, and its association with depression category was assessed.

**Results:**

A total of 326 consenting eligible women in the age group of 15–49 years were enrolled in the study of which 165 (50.6%) were currently using a hormonal contraceptive and 49 (15.0%) were using a nonhormonal contraceptive and the rest 112 (34.4%) were not using any contraceptives. There was no significant difference in the mean BDI scores (*p*=0.79) and degrees of depression (*p*=0.06) between the HC users and HC nonusers. However, individual symptoms of depression such as sadness (*p*=0.01), reduced libido (*p*=0.0002), feelings of pessimism (*p*=0.02), and failure (*p*=0.003) were found to be significantly higher in the HC users than non-HC users.

**Conclusion:**

We conclude that there was no significant difference in mean depression scores between groups. However, a few individual symptoms of depression were high in HC users suggesting depression as a potential side effect of hormonal contraceptive use.

## 1. Introduction

Oral contraceptive pills (OCPs) are the most commonly used contraceptive measure, owing to their effectiveness and practicability [1]. The estimated use of OCPs ranges from 45% [2] to 74.9% [3] followed by intrauterine devices at 69.9% in the Kingdom of Saudi Arabia (KSA). Women in this region were more familiar with oral contraceptive pill use than other ways of contraception [3]. Women often use oral contraceptive pills (OCPs) to prevent pregnancy or address menstrual symptoms. However, 32–60% of the women discontinue the use following 6 months for a variety of reasons, including mood changes [4].

Women are twice more likely to develop depressive disorder than men [5]. Incidentally, the prevalence of depression correlates with hormonal changes in women, especially during puberty, before menstruation, postpregnancy, and at perimenopause, suggesting hormonal fluctuations as a probable trigger for depression [6]. Female sex hormones have been hypothesized to play a role in causing depression [7]. There are several proposed mechanisms to explain how sex hormones affect mood. The first postulated theory suggests a decrease in catecholamines because of hormonal contraceptives (HCs)-induced monoamine oxidase (MAO) activation [8]. Second, decreased conversion of tryptophan to serotonin was suggested as a contributor to depression [9]. Third, HC is suggested to suppress vitamin B6 and B12 metabolism causing low serotonin and GABA [10]. Finally, HC dysregulates the HPA axis by elevating circulating cortisol levels, which cause effects analogous to chronic psychological stress and depression [11].

HC has mixed effects on mood, ranging from improved mood [12–14] to worse mood [15–20] or having no effect at all [21–23]. These findings suggest that some women may benefit from HC use, while others do not or even have a negative impact. Most studies have discovered a connection between teenage use of hormonal contraception and an increased risk of developing depression [14–16]. The impact of hormonal contraception on adult depression risk is less clear; some sources contend there is either no increased risk or a decreased risk in adults [12, 14, 22].

A Danish study of over a million women indicated that all hormonal forms of contraception increased the risk of depression across all age categories, with teens having the highest risk of depressive symptoms [16, 17]. The use of hormonal contraceptives was found to be positively associated with the use of any form of psychotropic drug in a Swedish survey of 800,000 women, although only among adolescents [17]. Another study of over 900,000 Swedish women found increased antidepressant use among adolescents on different types of OCPS, while in adults, this association was only observed with progestin-only pills [18]. The majority of studies, however, indicate that mood instability brought on by HC use is transient: Skovlund et al. showed that the largest correlations between HC use and depression occurred in the first 6–12 months after HC was started [16, 17].

Few randomized clinical trials revealed that OCP improved depressive symptoms while increasing mood swings and irritability [24, 25]. Literature shows mixed findings about the relationship of HC with depression. Therefore, we aimed to examine the association between HC use and concurrent depressive symptoms.

## 2. Materials and Methods

### 2.1. Study Design, Setting, and Population

This analytic cross-sectional study was conducted from November 2022 to August 2023 at Dr Soliman Fakeeh Hospital. The study was conducted after ethical approval (No-297/IRB/2022) from the institutional review board of Fakeeh College for Medical Sciences. Women in the reproductive age group, i.e., 15–49 years who visited the family planning unit of the obstetrics and gynecology department of the hospital, were the study population.

### 2.2. Sample Size and Sampling Strategy

Taking the prevalence (*p*) of depressive symptoms as 31.0% among women using hormonal contraceptives from a previous study from Saudi Arabia [26], at 95% confidence interval (*Z*) and taking the margin of error (*d*) as 5%, the sample size was calculated using the following formula:

(1) $$\begin{array}{c} \text{sample size},\;n=\text{DEFF}*Z_{1-\alpha /2}^{2}p\frac{\left(1-p\right)}{d2}, \end{array}$$

where DEFF=1, *Z*=1.96, *p*=0.33, and *d*=0.05.

The minimum sample size came out to be 326. Therefore, a total of 326 nonpregnant women in the reproductive age group of 15–49 years were included in the study by employing a consecutive sampling technique, in which every eligible woman who visited the clinic was included.

### 2.3. Eligibility Criteria

Inclusion criteria: Women in the reproductive age group of 15–49 years visiting the family planning unit of the hospital and giving written informed consent for participating were included.

Exclusion criteria: Women with a history of diagnosed clinical depression or use of psychiatric medication in the past, family history of depression, and chronic physical illness were excluded from the study.

### 2.4. Measurements

#### 2.4.1. Study Questionnaire

Data were collected using a pretested semistructured questionnaire consisting of sociodemographic characteristics, medical including obstetric, menstrual and contraceptive history, and Beck's depression inventory-II (BDI-II). Sociodemographic and medical characteristics assessed were age, marital status, level of education, socioeconomic status, no. of living children, history of any health problem in past 2 weeks, self-rating of health, smoking status, type and method of contraceptive currently used with duration, week of menstrual cycle, etc. Researchers in consultation with the experts prepared the questionnaire after reviewing similar related studies.

The Arabic version of Beck's depression inventory-II (BDI-II) was used to measure the outcome, i.e., depression or depressive disorder. The BDI-II is a widely used 21-item scale to assess depressive symptoms that can be used by a trained interviewer or self-rated [27]. BDI-II is used both in clinical practice and in research to assess the level of depression in patients and also in healthy persons with good validity and reliability. The items rating is on a 4-point Likert-type scale (0–3 points per item). The severity of symptoms is interpreted as “normal” (1–10), “mild” (11–16), “borderline clinical depression” (17–20), “moderate” (21–30), and “severe” (31–40) and extreme depression (>40) [27]. The Arabic version of BDI-II, used in the study, has been shown to have good construct validity and internal consistency [28].

The study questionnaire was pilot-tested on 30 participants to check for validity and reliability which yielded a Cronbach's alpha value of 0.83 implying good internal consistency. The data of the subjects recruited for the pilot study were excluded from the main study.

#### 2.4.2. Study Procedure

The survey was conducted at Dr. Soliman Fakeeh Hospital, Jeddah, Kingdom of Saudi Arabia (KSA), on a total of 326 nonpregnant women in the reproductive age group of 15–49 years who visited the family planning unit of the obstetrics and gynecology department of the hospital and came to the hospital pharmacy for a refill. Written informed consent was taken from all participants. The information about age, sociodemographic details, day of the menstrual cycle, and types of contraceptive used and duration of its use was recorded on the data collection sheet by the trained interviewers (6th-year medical students). Women currently using any hormonal contraceptive (HC) method, either oral or injectable, for the duration of one week or more were grouped as “HC users” and those using any nonhormonal contraceptive method and not using any of the contraceptives were categorized as “nonusers.” The interviewers administered Beck's depression inventory-II (BDI-II) to assess depression in the participants and give the appropriate score to denote its severity.

### 2.5. Statistical Analysis

Descriptive statistics were performed for the sample characteristics. The normality of the collected data was checked using Kolmogorov–Smirnov test. Continuous variables were summarized as means with their standard deviations for normally distributed data or medians with interquartile ranges in non-normally distributed data, whereas qualitative variables were summarized using frequency and percentages. The unpaired *t*-test was used to compare two independent continuous variables with normal distribution and the Mann–Whitney *U* test was used to compare two groups with non-normally distributed continuous variables. Chi-squared test/Fisher's exact test, a nonparametric test, was applied to compare two or more proportions. *p* value of less than 0.05 was taken as statistically significant.

## 3. Result

A total of 326 women in the reproductive age group of 15–49 years with no previous or family history of depressive disorder were included in the study. Women participants using hormonal contraceptives currently for the past one week or more were taken as “hormonal contraceptive (HC) users” and those using any nonhormonal methods and not using any contraceptives were taken as “HC nonusers.” Out of 326 women included, 165 were HC users and 161 were HC nonusers (nonhormonal = 49 and no contraceptives = 112). Their detailed sociodemographic and clinical profile is demonstrated in Table 1.

**Figure 1:**
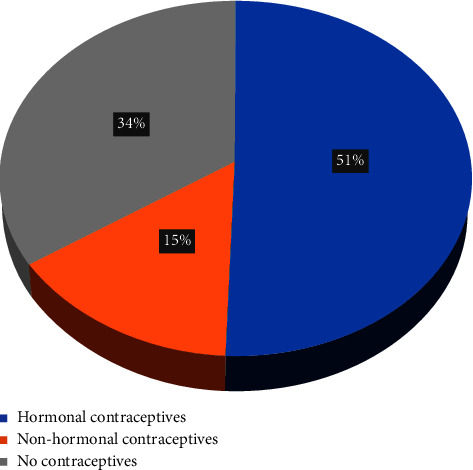
Distribution of the participants based on type of contraceptive method used (*n* = 326).

**Figure 2:**
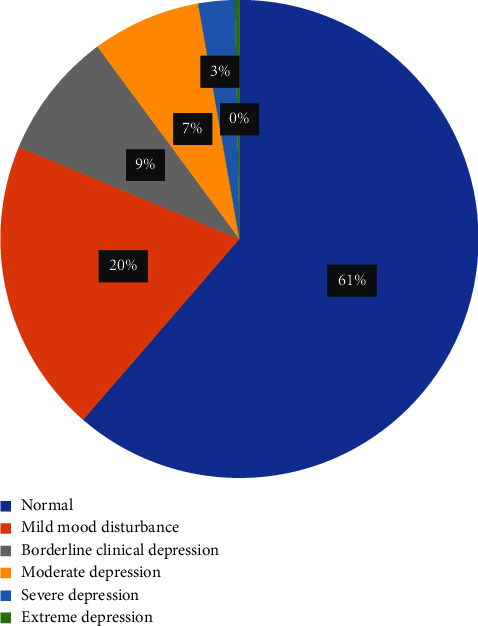
Distribution of the participants as per Beck's depression inventory depression categories (*n* = 326).

**Figure 3:**
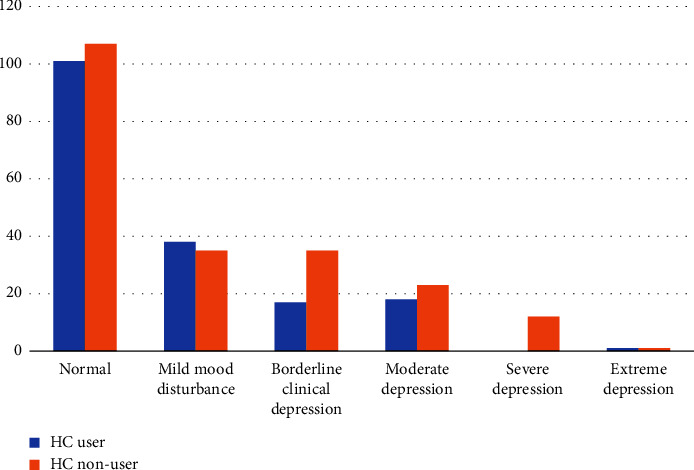
Comparison of degrees of depression between HC users and nonusers.

Of 326 participants, 51% used hormonal contraceptives, 34% were nonusers, and 15% used nonhormonal contraceptives. The overall prevalence of any method of contraception was 65.7% (*n* = 210) among the study participants. Among the sample, 50.6% (*n* = 165) reported hormonal contraception use, while 15.0% (*n* = 49) reported using nonhormonal methods (Figure 1). Among hormonal methods, 28.8% and 13.8% of the women reported using combined estrogen and progesterone and progestin-only pills, respectively, making OCPs the most used method. As for nonhormonal methods, copper IUDs ranked highest in percentage (9.2%) (Table 2). A total of 200 (61%) participants were normal, 65 (20%) had mild mood disturbances, 28 (9%) had borderline clinical depression, 24 (7%) had moderate, 8 (3%) had severe, and 1 (0.3%) had extreme depression (Figures 2 and 3). Participants who self-rated their health as poor have also shown significantly higher clinical depression (*p*=0.00001) (Table 3).

The mean Beck's inventory score of the participants was 9.86 ± 8.21 with scores ranging from 0 to 63 (Table 4). There was no discernible difference in mean Beck's depression inventory (BDI) score and degree of depression between the two groups (Table 4). A comparison of degrees of depression between HC users and nonusers is shown in Figure 2. Comparing oral pills and other hormonal contraceptives about depression status, no significant difference was found. Similarly, the duration of hormonal contraceptive use was also not found to be associated with the degree of depression (Table 5). However, the use of HC was associated with significantly more sadness (*p*=0.01), reduced libido (*p*=0.0002), feelings of pessimism (*p*=0.02), and failure (*p*=0.003) (Table 6).

## 4. Discussion

This study examined the association between the use of HC and depression among Saudi women in the reproductive age group. We found no significant difference in mean BDI score and degree of depression between the two groups. Our findings are consistent with several observational studies [15, 16, 23] and RCTs [25] which found no association between HC use and depression. On the contrary, in a 2013 study that used data from the National Longitudinal Study of Adolescent Health, OCP use shielded US women between the ages of 25 and 34 from depressive symptoms [12].

Our results are at odds with earlier studies that found using medication registry data that OCP use increased the likelihood of antidepressant use [17, 19]. Skovlund et al. [16] discovered in the Danish drug registry a considerable positive correlation between OCP usage and antidepressant use, especially among the youths. In their sample, current OCP users had an RR of antidepressant use of 1.8 in comparison with nonusers [16]. Skovlund et al. [16] looked at both antidepressant use and medical discharge coding for depression and discovered that there was a substantially weaker correlation between the two, indicating that antidepressant use is a poor proxy for an actual depression diagnosis. Both medical coding for depression and using antidepressants necessitate contact with a medical provider, leading to the potential risks of surveillance bias or confounding by indication.

Similarly, a Swedish study [17] reported that HC users had an adjusted OR of first-time use of psychotropic drugs of 1.34 with particularly high odds ratios (OR 3.36) in 12- to 14-year-old girls. Both these studies found a high risk of antidepressant consumption in teenagers and in those using progesterone-only HC [16, 17]. Interestingly, nonoral progesterone methods such as depot medroxyprogesterone acetate (DMPA) injection, skin patches, and intravaginal rings showed more depression when compared to oral progesterone pills [17, 18, 22].

A longitudinal cohort analysis of 740,000 women based on data from six Swedish Prescribed Drug Registers found no overall increased risk of depression, except a modest increase in risk in the adolescent age group. The major strengths of this study were its sample size, longitudinal design, adjustment for confounders like smoking, BMI, and past and family history of mental disorders. Additionally, recall bias was nonexistent as the data were based on ICD codes [22]. According to Anderl et al. [19], women who started using OCPs in their adolescence had a higher 1-year prevalence of depression than women who never used OCPs or who started using them in their adult years. This study had potential confounders such as recall bias and lack of information regarding the duration of use and type of OCP used.

Most studies found an increased risk of depression in the adolescent age group as compared to adults [16, 17, 22]. One convincing reason for the positive results could be attributed to confounders such as age at sexual debut, smoking, BMI, education, chronic health conditions, and social and behavioral factors. We believe that precocious sexual behavior and drug use are linked to an increased risk of taking OCP as well as an increased chance of developing depressive illness, which may affect these findings. It has been found that early sexual initiation is linked to increased risks of destructive behavior and poor mental health [29, 30]. Because HC use is a suitable proxy for an active sex life, the effect of starting HC at a young age may instead correspond to the effect of an early sexual debut. However, the increased risk of depressive symptoms among oral contraceptive users vanished after adjustments for sexual debut and smoking [23].

HC is used not just to prevent pregnancies but also to treat somatic symptoms like dysmenorrhea and premenstrual dysphoric disorder (PMDD), which may be more prevalent in adolescents and have a negative impact. In a cohort study of women in their 20s conducted in Australia, the connection between HC and depressive symptoms vanished when confounding factors such as noncontraceptive usage of HC were controlled [31].

Depression scores in our study did not vary with age, type of HC, and duration of HC use, unlike previous studies that have shown a high risk of depression in teenage and progesterone-only HC [16, 17]. The inclusion of solely married women in the sample could be an explanation for why we did not detect an increase in depression in our research. As sex among unmarried teenage girls was linked to an elevated risk of depression and suicidal behaviors [30], this exclusion could have lessened the confounding effect.

Our findings contradict regional studies that have found a higher frequency of depression in HC users than nonusers [26, 32]. One community-based cross-sectional study of 4853 participants reported higher depression in HC users while another conducted at primary healthcare centers also reported more depression in HC users [26]. Although our study did not find higher mean scores of depression in HC users, individual depressive symptoms such as sadness, feelings of pessimism and failure, and reduced libido were significantly high among the HC user group. Similarly, a previous study also reported higher scores of depressed mood and mood swings in users of combined oral contraceptive pills in comparison with placebo, and these mood changes were associated with altered reactivity in emotion circuits of the brain, especially affecting the insula and inferior frontal gyri and amygdala [33]. Another study demonstrated that mood worsening among individuals using OCPs was more likely in users with a history of depression [34]. This finding may indicate that a subset of depressed women may be more susceptible to mood worsening brought on by OCP due to their heightened sensitivity to the interplay between cycling gonadal steroids and affect. Estrogen regulates mood, and its withdrawal can lead to depression and anxiety as observed in conditions like PMDD and PPD [35]. The link between taking OCPs and depression may be attributed to the amount and type of progestogen contained in oral contraceptive pills. Progesterone increases GABA-induced inhibition of glutamate transmission and monoamine oxidase leading to decreased serotonin concentrations that can worsen mood symptoms [36]. However, our study excluded women with a history of depression and used any psychiatric drug in the past to reduce the confounding effect. Similar to our study, a few observational studies have suggested that HC users have diminished libido and sexual function [37], while RTCs showed no such link [38]. One may wonder why a feeling of pessimism and failure was associated more with HC users. This could be explained as secondary to the sad mood reported by the HC users or a personality attribute by itself.

### 4.1. Limitations

The cross-sectional design does not allow for mood assessment before pill consumption or identification of a depressive trend during the first several months of administration. There was no adjustment for selection bias and reporting bias. There is a possibility that those who stopped using hormonal contraceptives did not return for review and drug collection at the pharmacy further reducing the real estimate of depression. Moreover, the cross-sectional nature of the study cannot conclude a causal relationship.

Future prospective longitudinal studies should systematically document the short- and long-term effects of using different types of OCs (and other forms of hormonal contraceptives) on women's mental health; this may also help to identify further the specific biochemical mechanisms underlying the observed association.

## 5. Conclusion

We conclude that there was no significant difference in the mean BDI scores between the HC and non-HC user groups. However, there were significantly increased depressive symptoms such as sadness, decreased libido, and increased feelings of pessimism and failure in HC users. Our findings suggest that depression can be a potential side effect of HC use. Furthermore, longitudinal studies are required to warrant depression as an adverse effect of HC use.

## Figures and Tables

**Table 1:** Sociodemographic and medical characteristics of the study participants (*n* = 326).

Characteristics	Frequency *N*	Percentages
*Age groups (in years)*		
15–19	20	6.1
20–25	81	24.8
26–30	96	29.4
>30	129	39.6

*Socioeconomic status*		
Class I (<5000 SR)	47	14.4
Class II (5000–9999 SR)	86	26.4
Class III (1000–14999 SR)	84	25.8
Class IV (>15000 SR)	109	33.4

*Level of education*		
None	01	0.3
Elementary	04	1.2
Middle	14	4.2
High	70	21.4
Graduate	191	58.5
Postgraduate	46	14.1

*Marital status*		
Currently married	300	92
Widowed/divorced/separated	26	7.9

*No. of children*		
0	110	33.7
1	61	18.7
2	73	22.4
≥3	2	33.7

*Experienced any major life events (death of someone or any other major stressor) in last 6 months*		
Yes	172	52.7
No	154	47.2

*Smoking*		
Yes	93	28.5
No	278	85.2

Type of contraceptive used		
*Hormonal*	165	50.6
*Nonhormonal*	49	15.0
Not using any contraceptive	112	34.3

*Week of menstrual cycle*		
First	79	24.2
Second	100	30.6
Third	80	24.5
Fourth	67	20.5

**Table 2:** Type of hormonal and nonhormonal contraceptive methods used by the participants (*n* = 326).

Method	Specific type	Frequency	%
Hormonal	Combined oral pill	94	28.8
	Progesterone-only pill	45	13.8
	Injectable	04	1.2
	Patch	15	4.6
	Levonorgestrel-releasing IUD	02	0.6
	Progesterone implant	07	2.1
	Vaginal ring	07	2.1

Nonhormonal	Barrier methods	19	5.8
	Copper IUD	30	9.2

**Table 3:** Association of degree of depression with various sociodemographic and medical factors including contraception use (*n* = 326).

Variables	Depression category			*p* value^+^
	Normal (*n* = 200) *N* (%)	Mild mood disturbance (*n* = 65) *N* (%)	Borderline/moderate/severe/extreme depression (*n* = 61) *N* (%)	
*Age group (in years)*				
15–30	116 (59.2)	41 (63.1)	40 (65.6)	0.36
>30	86 (40.8)	24 (36.9)	21 (34.4)	

*Marital status*				
Currently married	183 (91.5)	60 (92.3)	57 (29.7)	0.88
Separated/Divorced/widowed	17 (8.5)	5 (7.7)	4(15.4)	

*Education level*				
Up to elementary school	2 (1.0)	1 (1.6)	02 (3.3)	0.68
Up to high school	50 (25.0)	16 (24.6)	18 (29.5)	
Graduate or above	148 (74.0)	48 (73.8)	41 (67.2)	

*Socioeconomic status*				
Class 1	26 (13.0)	8 (12.3)	13 (21.3)	0.06
Class 2	48 (24.2)	16 (24.6)	22 (8.3)	
Class 3	58 (29.1)	19 (29.2)	07 (11.5)	
Class 4	68 (33.9)	22 (33.8)	19 (31.1)	

*No. of children*				
1	36 (18.1)	12 (18.4)	13 (21.3)	0.77
2	46 (23.0)	15 (23.1)	12 (19.7)	
≥3	118 (58.9)	38 (58.5)	36 (59.0)	

*Suffered any health problem in past 2 weeks*				
Yes	72 (36.0)	24 (36.9)	38 (62.3)	0.0009^∗^
No	128 (64.0)	41 (62.1)	23 (37.7)	

*Smoking*				
Yes	48 (24.1)	16 (24.6)	19 (31.1)	0.52
No	152 (75.9)	49 (75.4)	42 (68.9)	

*Self-rating of health*				
Very good/good	168 (84.0)	54 (83.1)	25 (41.0)	<0.00001^∗^
Fair	27 (13.5)	9 (13.8)	27 (44.3)	
Poor/very poor	5 (2.5)	2 (3.1)	9 (14.7)	

*Use of any contraceptive method*				
Present	136 (68.0)	41 (63.1)	37 (60.7)	0.51
Absent	64 (32.0)	24 (36.9)	24 (39.3)	

*Method of contraception used*				
Hormonal	104 (52.0)	32 (49.2)	29 (47.5)	0.59
Nonhormonal/no contraceptive	96 (48.0)	33 (51.8)	32 (52.5)	

*Type of contraceptive method*				
Oral contraceptive pills (OCPs)	88 (44.0)	27 (41.5)	24 (39.3)	0.54
Other hormonal/nonhormonal/no contraceptive	112 (56.0)	38 (58.5)	37 (60.7)	

*Week of menstrual cycle*				
First	48 (24.9)	17 ()	14 (17.7)	0.09
Second	60 (32.9)	26 ()	14 (14.0)	
Third	46 (21.8)	11 ()	23 (28.7)	
Fourth	46 (21.8)	11 ()	10 (14.9)	

^+^Chi-square/Fisher's exact test. ^∗^Statistically significant.

**Table 4:** Comparison of depression status using Beck's depression inventory (BDI) score between HC users and HC nonusers.

Parameters	HC users (*n* = 165)	HC nonusers (*n* = 161)	*p* value
Mean BDI score	9.74 ± 7.91	9.97 ± 8.41	0.79^1^

*Degrees of Depression (BDI scores)*			
Normal/mild mood disturbance (0–16)	136 (82.4)	129 (80.1)	0.05^2^
Borderline/moderate (17–30)	28 (17.0)	24 (14.9)	
Severe/extreme (31–51)	1 (0.6)	8 (5.0)	

^1^Independent *t*-test. ^2^Chi-square test.

**Table 5:** Association of degree of depression with type and duration of contraceptives used (*N* = 214).

Variables	Depression status		*p* value^+^
	Normal/mild mood disturbance (*n* = 173) *N* (%)	Clinical depression (BDI score > 16) (*n* = 41) *N* (%)	
*Type of contraceptive used*			
Oral pills	115 (66.4)	24 (58.5)	0.33
Other hormonal/nonhormonal	58 (33.5)	17 (41.4)	

*Duration of contraceptive use*			
<1 month	37 (21.3)	5 (12.1)	0.68
1–6 months	46 (26.5)	6 (14.6)	
6–12 months	101 (58.3)	19 (46.3)	

^+^Chi-square test.

**Table 6:** Comparison of individual symptoms of Beck's depression inventory between HC users and nonusers (*N* = 326).

Symptom	HC users (*N* = 165)	HC nonusers *(N* = 161)	*p* value^+^
*Sadness*			
Absent (score = 0)	83 (50.3)	101 (62.7)	0.02
Present (score = 1, 2, or 3)	82 (49.7)	60 (37.3)	

*Pessimism*			
Absent (score = 0)	119 (72.1)	135 (83.8)	0.01^∗^
Present (score = 1, 2, or 3)	46 (27.9)	31 (19.3)	

*Failure*			
Absent (score = 0)	114 (69)	134 (83.2)	0.003^∗^
Present (score = 1, 2, or 3)	51 (30.9)	32 (19.8)	

*Pleasure*			
Absent (score = 0)	98 (59.3)	94 (58.3)	0.85
Present (score = 1, 2, or 3)	67 (40.6)	67 (41.6)	

*Guilt*			
Absent (score = 0)	85 (51.5)	68 (42.2)	0.57
Present (score = 1, 2, or 3)	80 (48.4)	93 (57.7)	

*Punishment*			
Absent (score = 0)	117 (70.9)	121 (75.1)	0.38
Present (score = 1, 2, or 3)	48 (29)	40 (24.8)	

*Disappointment*			
Absent (score = 0)	107 (64.8)	120 (74.5)	0.06
Present (score = 1, 2, or 3)	58 (35.1)	41 (25.4)	

*Self-blaming*			
Absent (score = 0)	104 (63)	96 (59.6)	0.52
Present (score = 1, 2, or 3)	61 (36)	65 (40.3)	

*Suicidality*			
Absent (score = 0)	152 (92.1)	149 (92.5)	0.88
Present (score = 1, 2, or 3)	13 (7.8)	12 (7.4)	

*Crying*			
Absent (score = 0)	111 (67.2)	123 (76.3)	0.06
Present (score = 1, 2, or 3)	54 (32.7)	38 (23.6)	

*Irritation*			
Absent (score = 0)	94 (56.9)	93 (57.7)	0.88
Present (score = 1, 2, or 3)	71 (43.03)	68 (42.2)	

*Social withdrawal*			
Absent (score = 0)	100 (60.6)	92 (57.1)	0.52
Present (score = 1, 2, or 3)	65 (39.3)	69 (42.8)	

*Decision making*			
Absent (score = 0)	109 (66.0)	14 (8.6)	0.66
Present (score = 1, 2, or 3)	56 (33.9)	47 (29.1)	

*Body image*			
Absent (score = 0)	110 (66.6)	108 (67)	0.93
Present (score = 1, 2, or 3)	55 (33.3)	53 (32.9)	

*Work difficulty*			
Absent (score = 0)	100 (60.6)	81 (62.1)	0.06
Present (score = 1, 2, or 3)	65 (39.3)	80 (49.6)	

*Insomnia*			
Absent (score = 0)	81 (49)	95 (59)	0.07
Present (score = 1, 2, or 3)	84 (50.9)	66 (40.9)	

*Fatigue*			
Absent (score = 0)	83 (50.3)	75 (49)	0.50
Present (score = 1, 2, or 3)	82 (49.6)	86 (53.4)	

*Loss of appetite*			
Absent (score = 0)	102 (61.8)	114 (70.8)	0.09
Present (score = 1, 2, or 3)	63 (38.1)	47 (29.1)	

*Weight loss*			
Absent (score = 0)	118 (71.5)	117 (72.6)	0.81
Present (score = 1, 2, or 3)	47 (28.4)	44 (27.3)	

*Worry about health*			
Absent (score = 0)	103 (62.4)	99 (61.4)	0.86
Present (score = 1, 2, or 3)	62 (37.5)	62 (38.5)	

*Loss of libido*			
Absent (score = 0)	90 (54.5)	120 (74.5)	0.0002^∗^
Present (score = 1, 2, or 3)	75 (45.4)	41 (25.4)	

^+^Chi-square test. ^∗^Statistically significant.

## Data Availability

The data that support the findings of this study are available on request from the corresponding author (SS). The data are not publicly available due to restrictions, e.g., their containing information that could compromise the privacy of research participants.

## Abbreviations

HCs: Hormonal contraceptives
OCP: Oral contraceptive pills.
