# Corrigendum to “Association of Hormonal Contraceptives with Depression among Women in Reproductive Age Groups: A Cross-Sectional Analytic Study”

**DOI:** 10.1155/ogi/9871215

**Published:** 2025-09-04

S. Sultan, MD. A. Bashar, R. M. Bazhair, et al., “Association of Hormonal Contraceptives with Depression among Women in Reproductive Age Groups: A Cross-Sectional Analytic Study,” *Obstetrics and Gynecology International* 2024 (2024): 7309041, https://doi.org/10.1155/2024/7309041.

In the article titled “Association of Hormonal Contraceptives with Depression among Women in Reproductive Age Groups: A Cross-Sectional Analytic Study,” the Disclosure section was omitted in error.

The Disclosure section is shown below:

**Disclosure**

The article has previously been published as a preprint—https://www.preprints.org/manuscript/202406.1510/v1.

We apologize for this error.

